# Supplementary figures and images for: Combined non-psychoactive Cannabis components cannabidiol and β-caryophyllene reduce chronic pain via CB1 interaction in a rat spinal cord injury model
Source: PLoS One. 2023 Mar 13;18(3):e0282920. doi: 10.1371/journal.pone.0282920 (PMC10010563; doi:10.1371/journal.pone.0282920)

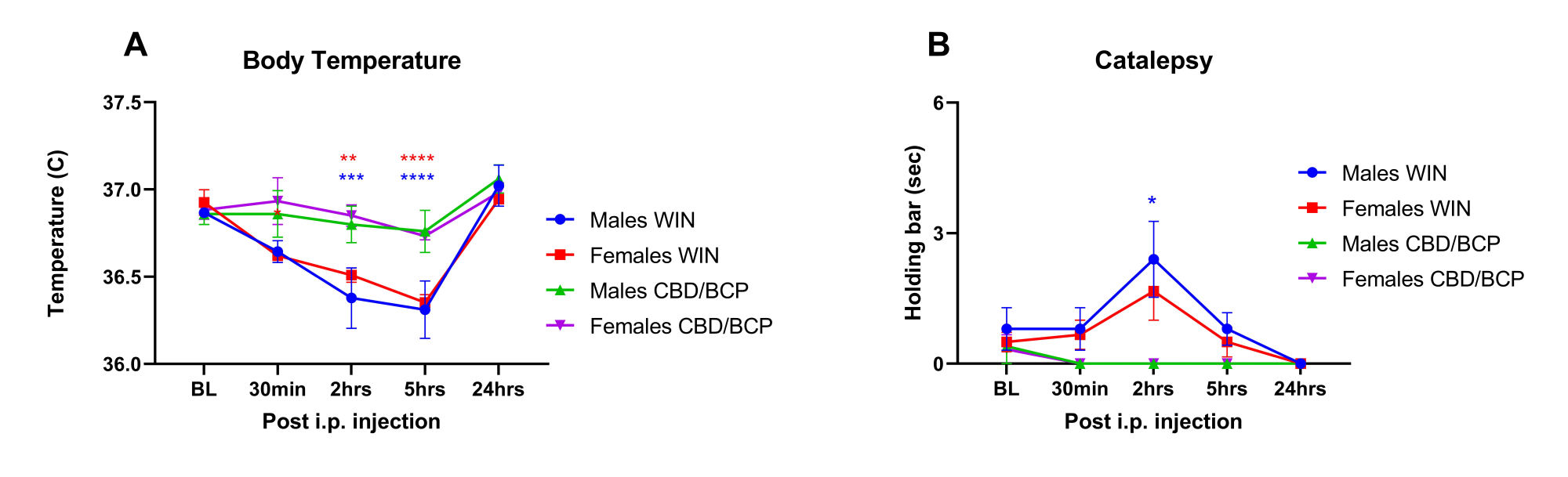

Supplement: S1 Fig — Time course curves showing the effect of maximum utilized antinociceptive dose combination of CBD (7 mg/kg) and BCP (35 mg/ kg) compared with WIN 55212–2 (3 mg/kg) on (A) body temperature and (B) catalepsy bar latency in males and females respectively (n = 6 per treatment group). Animals received a subcutaneous injection of WIN 55212–2 or an intraperitoneal injection and single oral administration of CBD:BCP following baseline measurements at 4 weeks post SCI surgery. *, **, ***, **** denote p < 0.05, 0.01, 0.001 and 0.0001 compared to baseline for each treatment group. (TIF) [file pone.0282920.s001.tif]

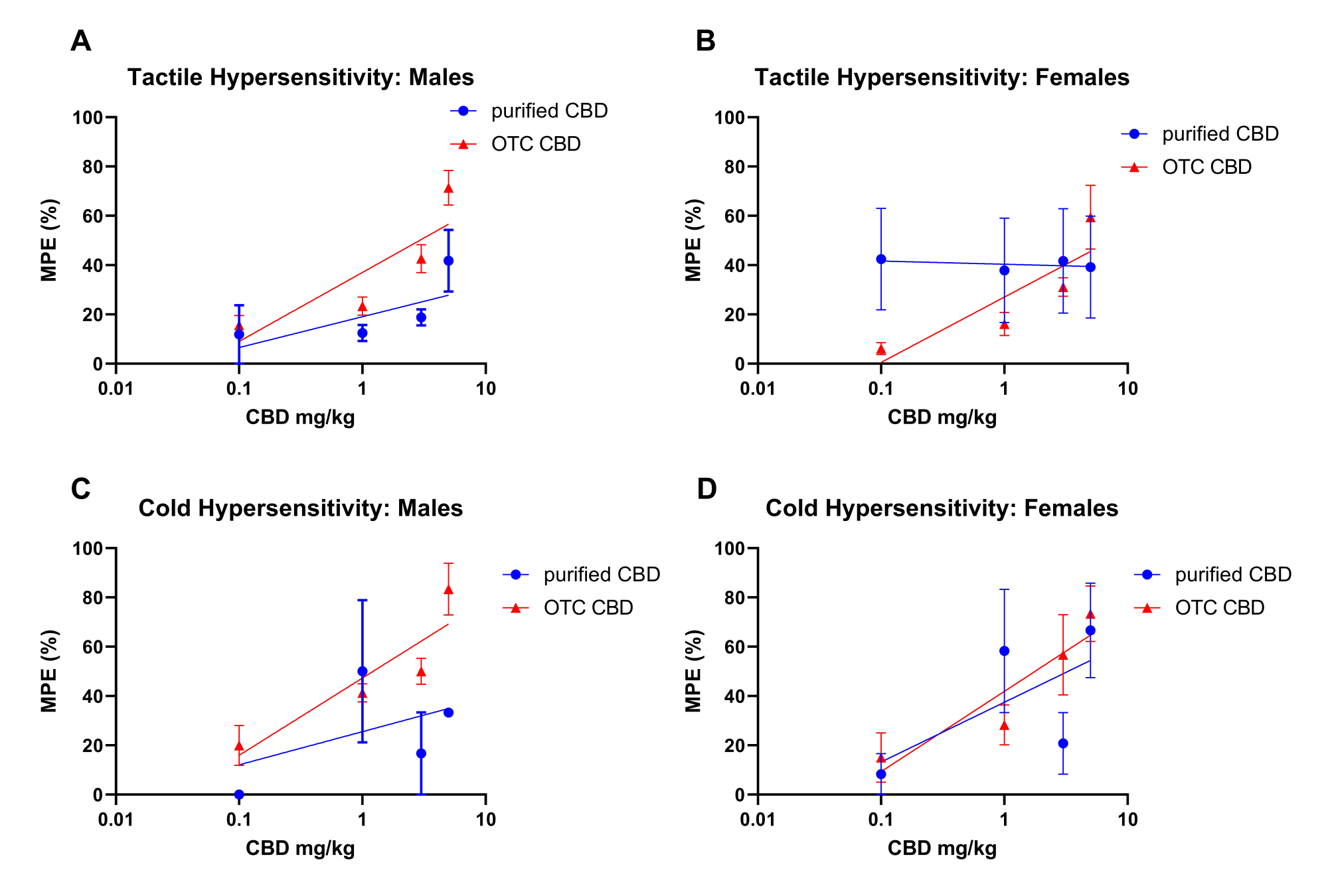

Supplement: S2 Fig — Dose response curves for the effect of CBD formulations on mechanical PWT in A) males and B) females and acetone responses in C) males and D) females. Data are shown as % maximal possible effect (% MPE) ± SEM. (TIF) [file pone.0282920.s002.tif]

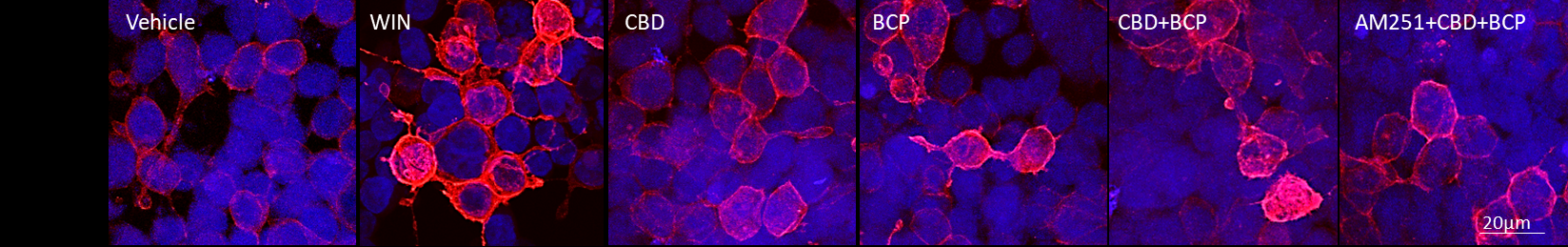

Supplement: S3 Fig — CB1 receptor is depicted in red, located in the cytoplasmic membrane when not activated, forming a rim around the cell. Upon activation receptor is translocated into cytoplasm in the form of small clusters with different density. Nuclei labeled with DAPI are blue. (TIF) [file pone.0282920.s003.tif]
