# Supplementary material for: Combined non-psychoactive Cannabis components cannabidiol and β-caryophyllene reduce chronic pain via CB1 interaction in a rat spinal cord injury model
Source: PLoS One. 2023 Mar 13;18(3):e0282920. doi: 10.1371/journal.pone.0282920 (PMC10010563; doi:10.1371/journal.pone.0282920)
Supplement: Attachment — Submitted filename: Response to Reviewers.docx [file pone.0282920.s004.docx]

Dear Dr. Mukhopadhyay,

We would like to thank you and the reviewers for the positive response and helpful suggestions for improving our manuscript. We have addressed the reviewer suggestions in the revised manuscript (marked in red) as follows:

Editor comment: *I would like to add a detailed discussion about the gender differences and possible hypothesis*.

Response: We have revised this section of the discussion to include more in depth consideration of our observed gender differences and possible underlying mechanisms (p22-24, highlighted in red). We have also done an extensive literature search in the field for insights and comparisons with our findings, but there are thus far little in published reports regarding gender differences in spinal cord injury (SCI) pain responses to cannabinoids. However, our data does suggest that there is some reduced effectiveness of cannabinoids in reducing SCI tactile hypersensitivity in females, both in response to CBD or BCP individually, and in particular in response to the CBD/BCP combination. The reductions in cold allodynia were essentially comparable in males and females. These differences have been clarified in the revision. Since the most robust distinction observed between males and females was in response to the combination CBD/BCP, both in potency and efficacy in reducing SCI tactile hypersensitivity, and our findings suggest that the antinociceptive effects of this combination are mediated via CB1 receptors, a hypothesis is that there are sex differences in the CB1/endocannabinoid system in response to injury, as suggested by Reviewer 1 (below). Although there are no published reports showing this per se, we have incorporated findings that may support this hypothesis, and certainly should be pursued in future studies.

Reviewer 1: Thank you for your supportive comments and interest, and insightful suggestions. We have addressed the major comments in the revised manuscript as follows:

1. *Antinociceptive effects of both BCP and BCP treatment as well as their combined administration represented less robust in females than males. What can be the possible explanation of this phenomenon? Is there any sex difference in the expression CB1 receptors in the dorsal segment of these rat’s lumbar spinal cord following SCI?*

Response: We have done an extensive search to determine whether there are reported sex differences in the expression of CB1 receptors in the spinal cord following SCI. Although this has not been reported, there are reported changes in both CB receptors and other components of the endocannabinoid system following SCI. There have been some sex differences reported in other models of chronic pain (neuropathic and inflammatory) in the endocannabinoid system that may provide additional insights. These points as well as a more comprehensive comparison between male and female responses to cannabinoids have now been included in a detailed section in the revised discussion (p22-24). The underlying mechanisms of these interesting differences, while beyond the scope of the current study, warrant further investigation going forward.

2. *BCP is thought to be a CB2 receptor agonist and had been shown to play an anti-inflammatory role in several tissue injury models via activating CB2 receptor signaling (please see PMIDs: 22326488; 27379721; 28107775). Interestingly, the authors found that administration of AM630 (a CB2 antagonist) barely affected the effects of combined CBD-BCP treatment (Fig.9). However, the current SCI model is not entirely associated with chronic tissue inflammation. Did the authors study the anti-inflammatory effect of the combined CBD-BCP treatment in another neuropathic pain model (e.g.: carrageenan-induced inflammatory pain model) that involves inflammation and activation of inflammatory leukocytes? Please provide some data using another inflammatory pain model.*

Response: We have emphasized this point and included the additional references above clearly indicating the BCP is generally considered as a CB2 receptor agonist in mediating anti-inflammatory and anti-nociceptive effects. The current SCI model appears to involve both neuropathic and inflammatory components, as anti-inflammatory mediators in spinal cord tissue and surrounding CSF are markedly increased following this injury as reported previously in our lab (Dugan et al., 2020, 2021). However, this is not likely to be the primary contributor to this SCI pain, as previous findings in our lab have shown that selective CB1 antagonists, but not selective CB2 antagonists, block the analgesic effects of synthetic mixed CB1/CB2 agonists WIN 55,212-2or CP 55,940 in this model (Hama and Sagen, 2007; Hama et al., 2014). Thus, there is likely an additional contributing mechanism in the current observed robust effects of the combined CBD-BCP treatment. In addition, anti-inflammatory agents have only modest beneficial effects in reducing clinical chronic SCI pain. With regard to data in other neuropathic pain models with inflammatory components, while outside the scope of the current study, we have recently found a reduction in a phantom limb pain model by CBD/BCP combined administration, along with reduced spinal inflammatory markers – however this work is in progress and has been presented in abstract form (Eeswara et al., 2022). These points have been added to the revised discussion (p25-26).

3. *Did the authors investigate the CB1-dependence of CBD and BCP drugs when given separately? In the combined treatment setting, can the drugs facilitate allosterically one another’s effect on CB1 receptors?*

Response: Since the original goal of the study was to determine whether the combined administration of two reported mechanistically distinct readily available Cannabis components could provide increased attenuation of SCI-related neuropathic pain, we had relied on the literature reports for individual mechanisms. Thus, CB1-dependence of CBD and BCP effects were not originally anticipated. This caveat in retrospect is now included in the revised manuscript. However, we were also intrigued after obtaining this unexpected result indicating CB1 involvement in the combined CBD-BCP effect, and thus had initiated pilot in vitro internalization assays to indicate whether this may involve a direct effect on CB1 receptors. Using CB1 receptor expressing cells, results suggested that CBD alone produces little effect, and BCP alone produces marginal CB1-activated receptor internalization, while the combination of CBD and BCP appears to enhances CB1 internalization, and this is blocked by CB1 antagonist AM251. Thus, it is possible that the drugs can allosterically facilitate one another’s effect on CB1 receptors, as suggested. It must be emphasized that these are preliminary data at this stage and needs to be further explored in future studies. We have added examples of this pilot study as an additional supplementary figure (p26-37, Supplemental Figure 3).

Reviewer 2: Thank you for your positive comments suggested clarifications to enhance the readability of our manuscript. We have addressed the comments in the revised manuscript as follows:

2. *Abstract: 'Minimal cannabinoidergic adverse side effects were observed with high doses of the combination.' Write either adverse effects or side effects.*

Response: The sentence has been updated according to the suggestion (p2, L37)

3. *'A50' has not been defined in the manuscript as well as in the main text. It would be difficult for someone who is not familiar with this term*.

Response: We have added an explanation for “A50” into Experimental Design (p7, L146-147).

4. *The section 'Drugs administration' should be ahead of 'Experimental design'.*

Response: The section has been moved as suggested (p6)

5. *Results: Rewrite the title of each result in order to highlight the main message of that particular results*

Response: The titles have been re-written (highlighted in red font).

6. *How much the efficacy or analgesic potential of CBD and BCP alone and/or in combination can be compared with the approved analgesics (central and/or peripheral)? The authors need to report few experiments in the same model in order to answer this question?*

Response: We have utilized this model in a number of analgesics testing over the past several years, and clinically approved agents for SCI pain were originally tested in the pharmacologic validation of the model (Hama and Sagen, 2007). In particular, gabapentin, which is widely used as a first line treatment for neuropathic pain including SCI, results in dose-related attenuation of tactile hypersensitivity in this model, with effects of the highest dose (100 mg/kg) comparable to the combined CBD/BCP 3:22 dose (Hama and Sagen, 2007). Morphine is also comparatively effective at 3 mg/kg, but develops rapid tolerance and has high misuse and side effects risks (Hama and Sagen, 2009). These comparisons have now been added to revised results in the appropriate section (p15).

Thank you again for the positive reviews of our manuscript. We feel that the suggested reviewer revisions have improved our manuscript and are looking forward to its publication in PLOS ONE.
