# Supplementary material for: Combined non-psychoactive Cannabis components cannabidiol and β-caryophyllene reduce chronic pain via CB1 interaction in a rat spinal cord injury model
Source: PLoS One. 2023 Mar 13;18(3):e0282920. doi: 10.1371/journal.pone.0282920 (PMC10010563; doi:10.1371/journal.pone.0282920)
Supplement: Attachment — Submitted filename: Response to Reviewers2.docx [file pone.0282920.s005.docx]

Dear Dr. Mukhopadhyay,

We would like to thank you and the reviewers again for positive comments and suggestions. We have addressed the reviewer suggestions in the revised manuscript as follows:

Reviewer #1: The authors have improved the present manuscript considerably by incorporating suggestions and remarks by the reviewers. The figures have been adequately revised in order to meet with the general concept of the study. There is only one major comment though that need to be addressed:

1. A detailed descriptions is needed for the cell culture experiments in the materials and methods section explaining the cell type as well as the duration of treatment used for studying CB1 receptor internalization following combined CBD/BCP administration as well as the imaging technique. Based on the corresponding figure legend, the authors used a CB1 receptor expressing cell line, however, receptor internalization was revealed in a non-specific manner (labelled hemagglutinin). Due to the lack of reliable antibodies, the authors should use other approach (e.g.: GFP-tagged CB1 receptor expressing cell line) to provide mechanistic explanation on the synergistic effects of CBD and BCP on CB1 receptor internalization.

Thank you for the suggestion, we have added CB1 cell culture and internalization assay methods into Method section (lines 237-261).

The HEK293 cell line used in this section express CB1 receptor with an N-terminus hemagglutinin (HA) tag; therefore the HA antibody is specific for labeling the tagged CB1 receptor and tracking the internalization of the receptor. This approach was developed for this purpose as reported by other studies (e.g. Daigle et al., 2008; ref 54; Grimsey et al., 2008; ref 56) and has been used in previous work in our lab (Jergova et al., 2021; ref 57) as well. Although the current study included CB1 internalization as a supplemental pilot assessment, future work will be undertaken to further understand the cellular mechanisms underlying CBD and BCP synergism.

Thanks to all reviewers for the positive reviews of our manuscript.
